# Chk1 inhibition as a novel therapeutic strategy in melanoma

**DOI:** 10.18632/oncotarget.25765

**Published:** 2018-07-13

**Authors:** Bor-Jang Hwang, Gautam Adhikary, Richard L. Eckert, A-Lien Lu

**Affiliations:** ^1^ Department of Biochemistry and Molecular Biology, University of Maryland School of Medicine, Baltimore, MD, USA; ^2^ University of Maryland Greenebaum Comprehensive Cancer Center, Baltimore, MD, USA; ^3^ Department of Dermatology, University of Maryland School of Medicine, Baltimore, MD, USA; ^4^ Department of Reproductive Biology, University of Maryland School of Medicine, Baltimore, MD, USA

**Keywords:** BRAF, Chk1, inhibitors, drug resistance, melanoma

## Abstract

Melanoma patients respond poorly to chemotherapies because they acquire drug resistance. Therapies that can overcome the resistance to inhibitors of the mutated BRAF protein kinase in melanoma are urgently needed. Chk1 protein kinase is a central component of the DNA damage response and plays a crucial role in controlling cell cycle progression. Analyses indicate that low mRNA expression of Chk1 is significantly associated with good overall survival of melanoma patients. To evaluate the effectiveness of Chk1 inhibitors in melanoma therapy, we have generated BRAF inhibitor (PLX4032 or vemurafenib) resistant melanoma cell lines (A375-PLX-R and WM9-PLX-R) from A375 and WM9, respectively. We observe that AKT (protein kinase B) is constitutively activated in A375-PLX-R, but not in WM9-PLX-R cells, suggesting that these cells develop resistance to PLX4032 through different mechanisms. We show that a potent and specific inhibitor of Chk1 (PF477736) is effective in reducing cell viability and colony formation of PLX4032-resistant cells. Even more impressively, PF477736 triggers PLX4032-resistant melanoma cells to regain sensitivity to the PLX4032. Mouse xenograft studies show that treating A375-PLX-R derived tumors with combined PLX4032 and PF477736 significantly reduce tumor growth. Combined treatments with PLX4032 and PF477736 reduce the levels of total Chk1 protein and alter Chk1 phosphorylation at several sites in both PLX4032 sensitive and resistant melanoma cells. Combinatorial treatments with PLX4032 and PF477736 to melanoma cells substantially induce DNA damage and cell death. Our results suggest that Chk1 inhibitors may provide new therapy options for melanoma patients.

## INTRODUCTION

Melanoma is the deadliest form of skin cancer and has a strong tendency to metastasize with a poor prognosis for survival [1]. The conversion of melanocytes to melanoma cells is frequently accompanied by alterations in key signaling pathways [2]. The Ras pathways are activated by many different stimuli through binding to the membrane receptors [3]. Ras then activates RAF (BRAF and CRAF) and PI3K (phosphatidylinositol 3-kinase). RAF kinases phosphorylate MEK (mitogen-activated protein kinase) which then activates ERK (extracellular signal-regulated kinase). PI3K promotes while PTEN (phosphatase and tensin homology) inhibits the activities of downstream PDK1 (3-phosphoinositide-dependent protein kinase-1) and AKT (or PKB, protein kinase B). Activation of these pathways leads to cell proliferation and survival. Approximately 60% of melanomas contain a V600E mutation in the *BRAF* gene [4, 5]. Constitutive activation of the ERK pathway caused by BRAF^V600E^ mutation accompanied by loss of PTEN tumor suppressor is the most common cause of melanomagenesis [4, 6]. Targeted therapy against BRAF mutation represents one of the most significant advances in the treatment of melanoma (reviewed in [7]). Vemurafenib (PLX4032), a specific BRAF inhibitor (BRAFi), has been approved to treat late-stage melanoma with BRAF^V600E^ mutation [8]. While PLX4032 targets melanoma with high efficacy and selectivity, the duration of response is usually limited (about 6 months) [7, 9, 10]. Thus, novel strategies to treat BRAFi-resistant melanoma are urgently needed.

Chk1 kinase is a central component of the DNA damage response and plays a crucial role in controlling cell cycle progression [11]. The DNA damage response pathway is activated to elicit both DNA repair processes and cell cycle arrest (which allows time for DNA repair). When DNA damage is extreme, apoptosis is triggered [11, 12]. Chk1 phosphorylation at S317 and S345 by ataxia telangiectasia and Rad3-related protein (ATR) is essential for cell-cycle checkpoint control [13, 14]. During DNA damage response, Chk1 autophosphorylation at S296 after phosphorylation by ATR [15, 16] is critical for cell cycle arrest [17]. Recent studies have shown that Chk1 can be phosphorylated by CDK and AKT at different residues, affecting subcellular localization [17, 18]. At G0/G1 transition, Chk1 is phosphorylated at S280 by Ras/mitogen-activated 90-kDa ribosomal S6 kinase (p90 RSK) [19] and translocated from the cytoplasm to the nucleus. However, in response to DNA damage during the G2 phase, Chk1 phosphorylation at S280 by AKT reduces nuclear localization and impairs DNA damage response [20–22]. Cell cycle checkpoints are promising targets for anticancer therapies because they control cancer cell responses to anticancer agents [23, 24]. Chk1 inhibitors (Chk1i) have emerged as very effective therapeutic agents alone and in combinatorial therapies [25–29]. PF477736, a potent and specific inhibitor of Chk1 (with ≈100-fold selectivity over Chk2) [28, 30], potentiates the antitumor activity of gemcitabine [30] and is in phase 1 clinical trials with gemcitabine [23, 24]. In this report, we find that PF477736 significantly retards melanoma cell growth, but even more impressively, triggers PLX4032-resistant melanoma cells re-sensitizing to PLX4032. We suggest that Chk1i may prevent the development of BRAFi resistance in melanoma because Chk1 inhibition can cause cancer cells to arrest improperly with damaged DNA and undergo apoptosis.

## RESULTS

### Chk1 is a biomarker of melanoma prognosis

**Figure 1:**
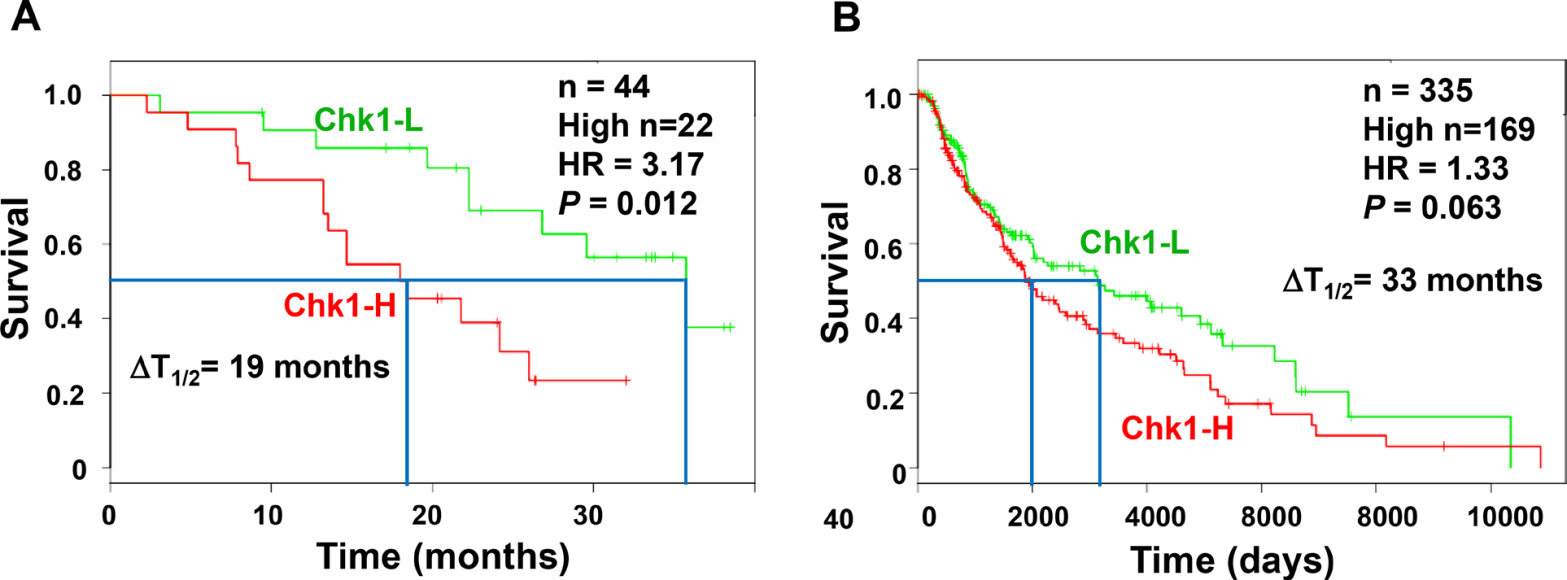
High Chk1 expression is associated with poor prognosis of melanoma The Kaplan–Meier plots for the survival of melanoma patients are constructed from an online database [35] using Chk1 mRNA expression as a marker. (**A**) There are 44 melanoma patients in the Bogunovic database and 22 have high Chk1 mRNA expression. We observed that low mRNA expression of Chk1 is significantly associated with good overall survival of melanoma patients [hazard ratio (HR) = 3.17; *P* = 0.012]. The 50% survival time (T_1/2_) of low Chk1 expression patients is ≈19 months longer (ΔT_1/2_) than that of high Chk1 expression patients. (**B**) Total melanoma patients in the SKCM-TCGA database are 335 with 169 having high Chk1 mRNA expression. Low mRNA expression of Chk1 is also associated with good overall survival of melanoma patients [HR = 1.33; *P* = 0.063; ΔT_1/2_ ≈33 months].

Chk1 kinase is required to manage DNA repair, DNA replication, and cell cycle progression in cancer cells [11, 31]. Several Chk1i have been demonstrated to reduce the cell viability of melanoma cells [32–34]. To examine whether Chk1i are effective for melanoma patients, we analyzed the survival of melanoma patients from an online database [35] using Chk1 mRNA expression as a marker. By analyzing 44 melanoma patients of the Bogunovic data set, we observed that low mRNA expression of Chk1 is significantly associated with good overall survival of melanoma patients [hazard ratio (HR) is 3.17; *P* = 0.012] (Figure 1A). The 50% survival time of low Chk1 expression patients is ≈19 months longer than that of high Chk1 expression patients. Analysis of 335 melanoma patients in the SKCM-TCGA data set also indicates that low mRNA expression of Chk1 is associated with good overall survival of melanoma patients (HR is 1.33; *P* = 0.063) (Figure 1B). The 50% survival time of low Chk1 expression patients is ≈33 months longer than that of high Chk1 expression patients. These results have implications for employing Chk1 inhibitors in melanoma treatment.

### Development of PLX4032-resistant melanoma cell lines

To compare the effectiveness of Chk1 inhibitors on different melanoma cells, we generated BRAF inhibitor PLX4032 resistant melanoma cell lines (A375-PLX-R and WM9-PLX-R) from A375 and WM9 respectively. These cell lines are p53 positive, but have different expression of the PTEN tumor suppressor. A375 cell line is PTEN-positive while WM9 cell line does not express PTEN protein due to homozygous deletion of the *PTEN* gene [36]. PTEN has been shown to play a direct role in DNA repair [37, 38] and DNA damage response [39] through interaction with Chk1 and the tumor suppressor p53. Thus, PTEN status may influence cellular response to Chk1 inhibitors.

**Figure 2:**
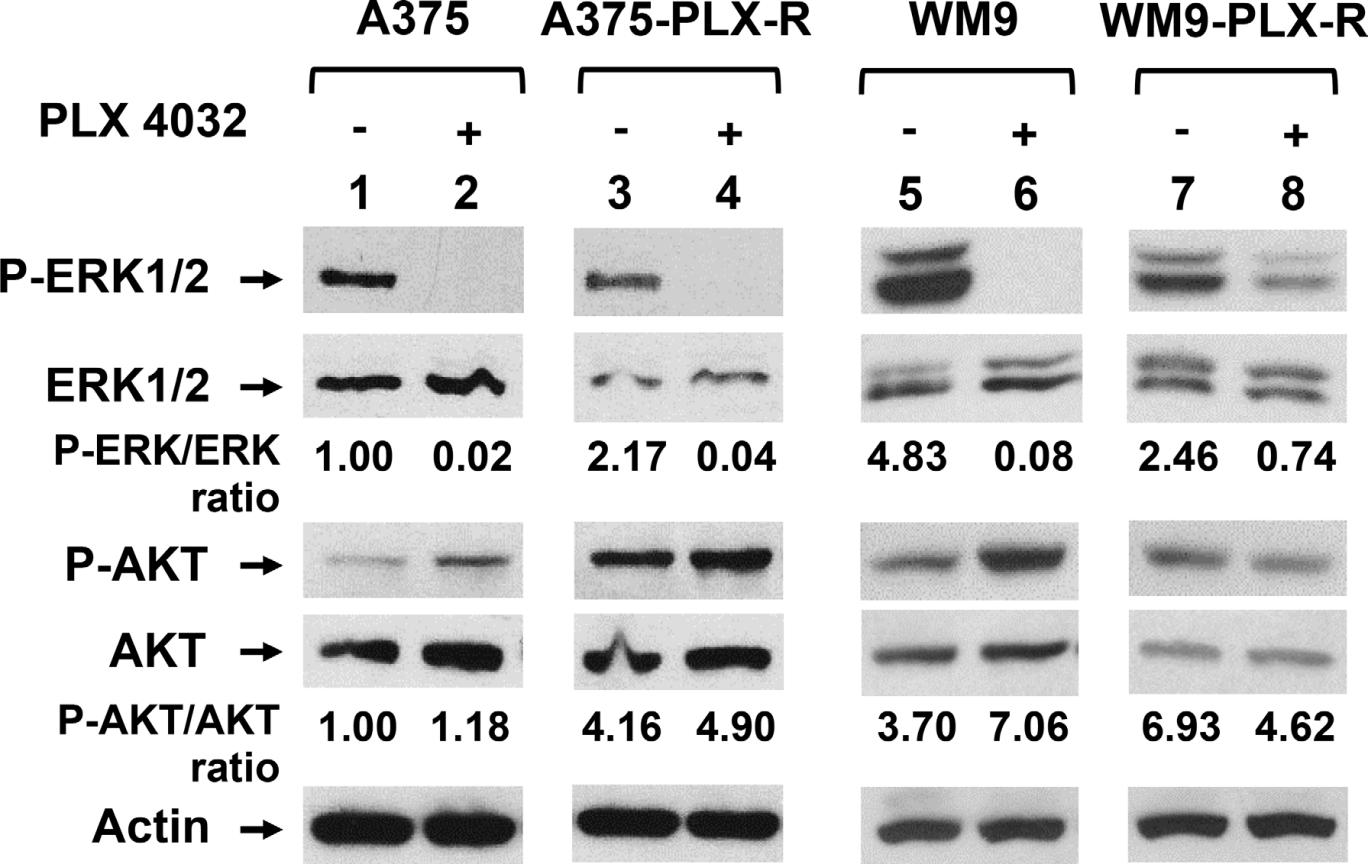
PLX4032-resistant melanoma cells have altered Ras signal pathways A375-PLX-R and WM9-PLX-R are derived from A375 and WM9, respectively. Cells were treated with 7.5 μM PLX4032 for 6 h or left untreated. Cell extracts were prepared and about 25 μg of total protein were separated on 10–16% SDS-polyacrylamide gels and transferred to nitrocellulose membranes for Western blotting analysis with respective antibodies. The images were quantitated as described in MATERIALS AND METHODS. Ratios of phosphorylated proteins (P-ERK1/2 and P-AKT) over total proteins were calculated and then adjusted relative to the value of lane 1 (untreated A375 cells). Values of relative ratios presented are the averages of images from two independent experiments.

The A375-PLX-R cells have been shown to have altered Hippo pathways [40]. Because melanoma can develop BRAFi resistance by activating alternative survival pathways [7, 9, 10], we examined whether the Ras pathways are altered in A375-PLX-R and WM9-PLX-R cells. The ratio of P-ERK/total ERK is increased in untreated A375-PLX-R (Figure 2, compare P-ERK/ERK in lanes 1 and 3). However, the ratio of P-ERK/total ERK is decreased in untreated WM9-PLX-R (Figure 2, compare P-ERK/ERK in lanes 5 and 7). AKT is constitutively activated in untreated A375-PLX-R (Figure 2, P-AKT in lanes 1 and 3), but not in untreated WM9-PLX-R cells as compared to their parental cell lines (Figure 2, P-AKT in lanes 5 and 7). We then analyzed the alterations of the Ras pathways by treating the cells transiently with 7.5 μM PLX4032 for 6 h which did not lead to massive cell death. As expected, phosphorylated ERK1/2 is absent in A375 and WM9 cells (Figure 2, lanes 2 and 6 for P-ERK1/2) following PLX4032 treatment. Treated A375-PLX-R cells also have no detectable phosphorylated ERK1/2 (Figure 2, P-ERK1/2 in lane 4), but treated WM9-PLX-R cells contain ≈32% level of phosphorylated ERK1/2 as compared with untreated cells (Figure 2, compare lanes 7 and 8 for P-ERK/ERK ratio). The ratio of P-AKT/total AKT is unchanged in PLX4032-treated A375 and A375-PLX-R cells (Figure 2, compare P-AKT/AKT in lanes 1–4). The ratio of P-AKT/total AKT is increased in PLX4032-treated WM9, but decreased in treated WM9-PLX-R cells (Figure 2, compare P-AKT/AKT in lanes 5–8). These data suggest that these two PLX4032-resistant cell lines develop resistance to PLX4032 through different mechanisms.

### PF477736 is an effective therapeutic agent to melanoma cells

**Figure 3:**
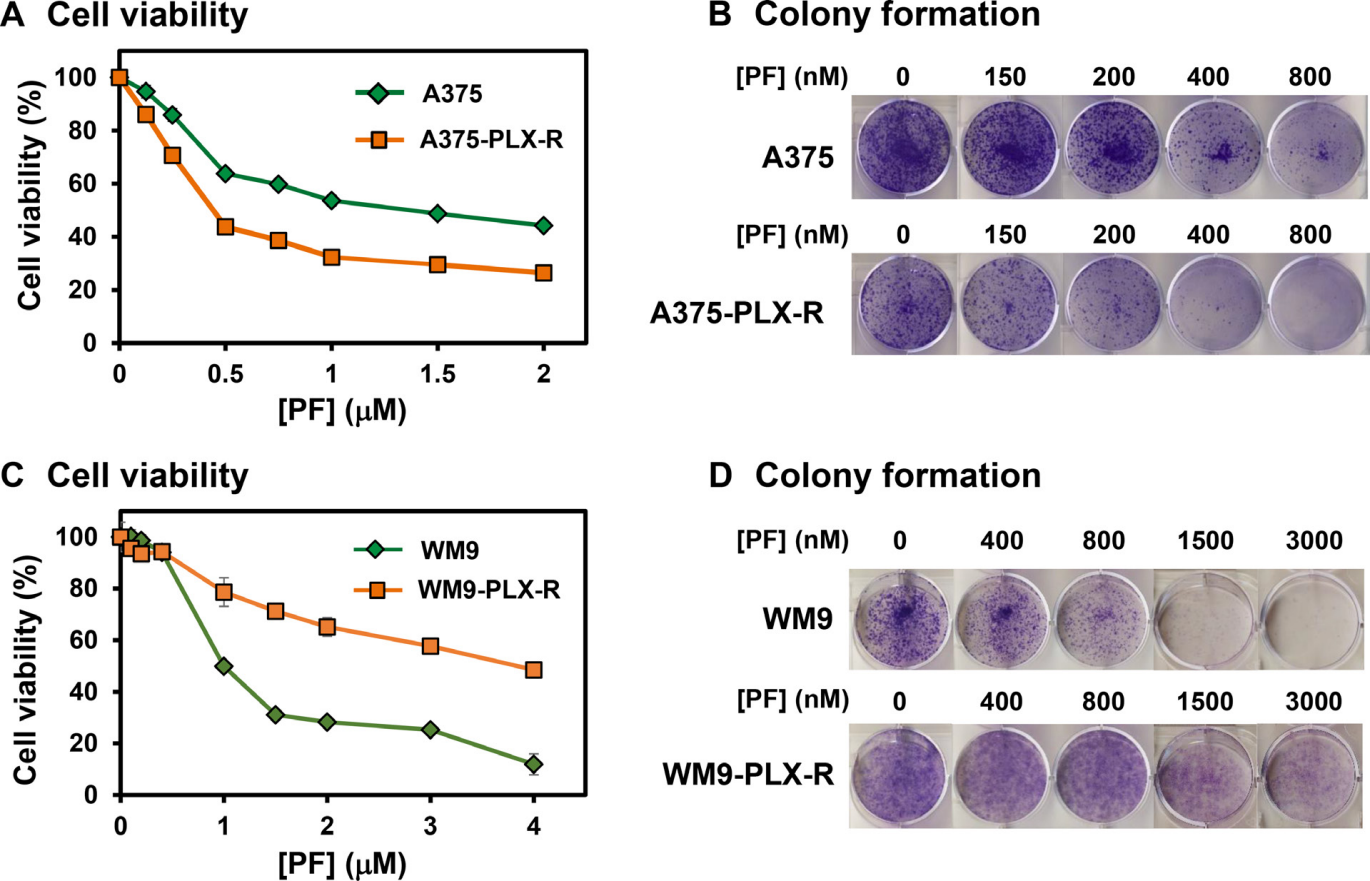
Response of melanoma cells to Chk1 inhibitor PF477736 (**A**) and (**B**) A375-PLX-R melanoma cell is more sensitive to PF477736 than A375 in both cell viability and colony formation assays. (**C**) and (**D**) WM9-PLX-R melanoma cell is less sensitive to PF477736 than WM9 in both cell viability and colony formation assays. One-day post-seeding, the cells were treated with various concentrations of PF477736 or DMSO for 48 h. Cell viability and colony formation were measured as described in MATERIALS AND METHODS. The error bars reported in (A) and (C) are the standard deviations of the averages from triplicates of three independent experiments.

We then examined the response of both A375 and A375-PLX-R melanoma cells to a potent Chk1 inhibitor (PF477736) which has higher specificity over Chk2 (IC_50_ = 0.49 nM for Chk1 and IC_50_ = 47 nM for Chk2) [28, 30] and has been in phase 1 clinical trials with gemcitabine [23, 24]. Figure 3A and 3B show that both cell lines are sensitive to Chk1i. PF477736 is more effective in inhibiting cell viability (Figure 3A) and colony formation (Figure 3B) of A375-PLX-R than A375 cells (IC_50_ of viability for A375 and A375-PLX-R are 1.4 and 0.4 μM, respectively). These results suggest that Chk1 function is more important for the cell survival of A375-PLX-R than A375 cells.

We then tested whether the effect of Chk1i could be extended to PTEN-negative WM9 and its derived PLX4032-resistant cells. Figure 3C and 3D show that both cell lines are also sensitive to Chk1i. However, PF477736 is more effective in inhibiting cell viability (Figure 3C) and colony formation (Figure 3D) of WM9 than WM9-PLX-R cells (IC_50_ of viability for WM9 and WM9-PLX-R are 1.0 and 4.0 μM, respectively). Thus, in contact to A375 cell lines, Chk1 function is less important for the cell survival of WM9-PLX-R than WM9 cells.

### PF477736 re-sensitizes PLX4032-resistant melanoma cells to PLX4032

**Figure 4:**
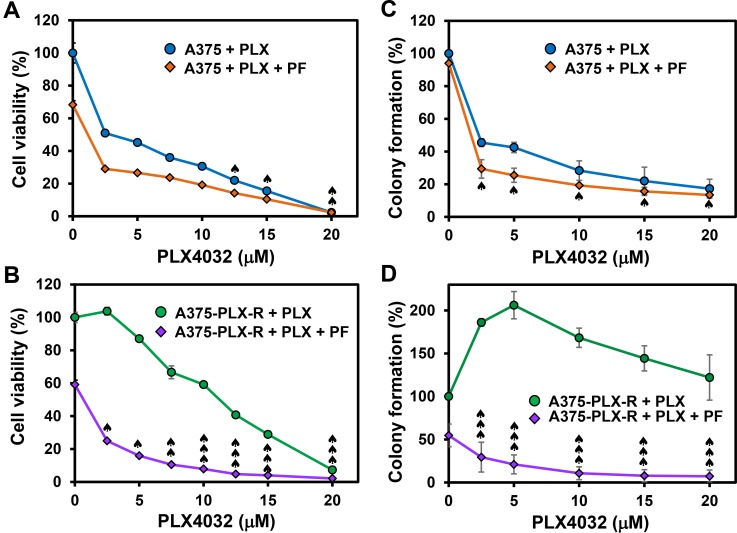
Chk1 inhibitor PF477736 re-sensitizes PLX4032-resistant melanoma cells to PLX4032 The melanoma cells were treated with Chk1i (PF477736, 0.15 μM) in combination with increasing concentrations of BRAFi (PLX4032, 0–20 μM) as indicated on the X-axis. Cell viability (**A** and **B**) and colony formation (**C** and **D**) were measured as described in MATERIALS AND METHODS. A375-PLX-R cells form more colonies in the presence of 2.5–15 μM PLX4032 than in the absence of PLX4032 (Figure 4D, green curve). The error bars reported are the standard deviations of the averages from triplicates of three independent experiments. Combination index (CI) for cells treated with two drugs was determined by CompuSyn software for Chou and Talalay analysis as described [54]. One, two, and three ♠ represent CI (0.5–0.9), CI (0.25–0.5), and CI (<0.25), respectively.

**Figure 5:**
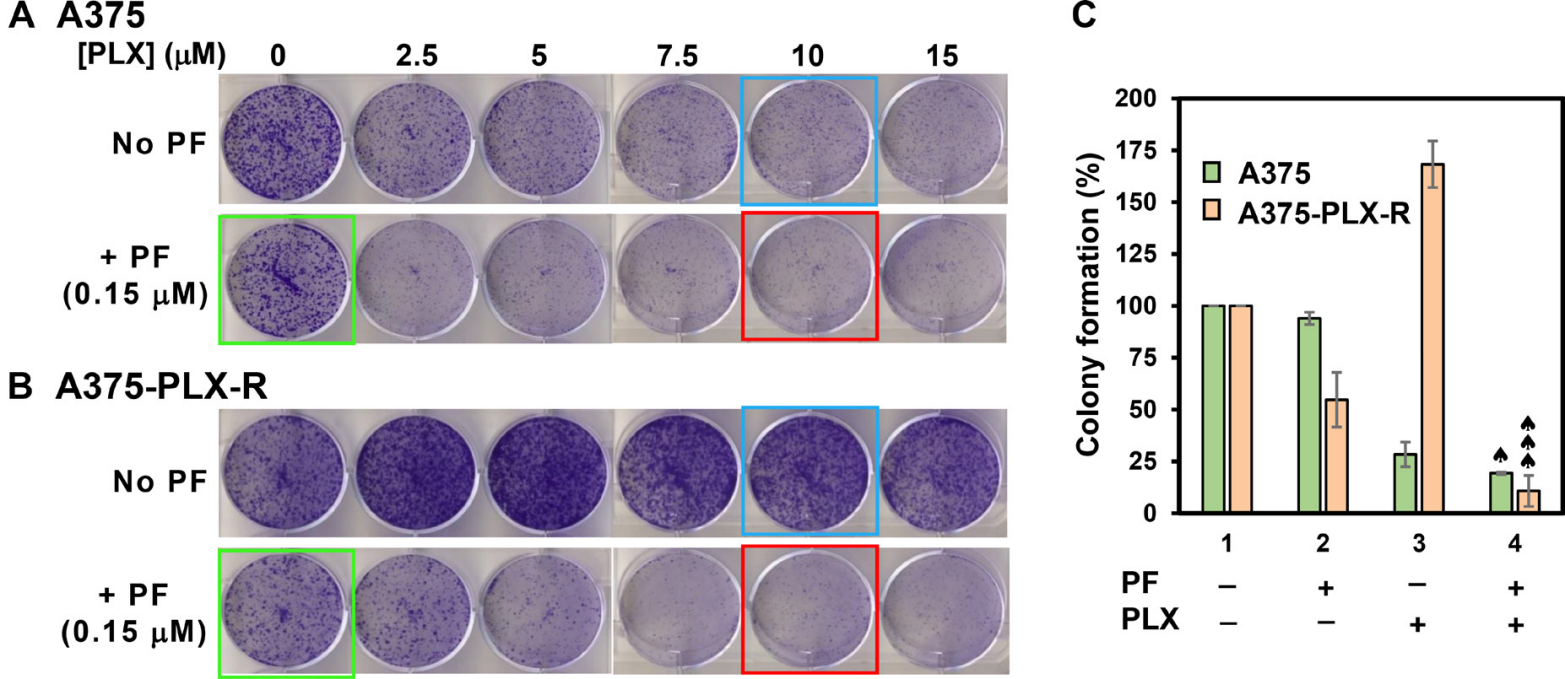
Colony formation of A375 (**A**) and A375-PLX-R (**B**) cells in the presence of BRAFi PLX4032 and/or Chk1i PF477736. Top panels in both (A) and (B) are cells treated with different concentrations of PLX4032 alone as indicated and bottom panels are cells treated with respective PLX4032 concentrations plus 0.15 μM PF477736 for 2 days or left untreated. The cells were recovered in regular media for 10 days and colonies were counted. Data from more than three experiments with 10 μM of PLX4032 (blue boxed), 0.15 μM of PF477736 (green boxed), and PLX4032+PF477736 (red boxed) are shown in (**C**). The error bars, CI calculation, and ♠ symbol were similar to those as described in Figure 4.

The response to PLX4032 is not durable because many patients acquire drug resistance [9, 10]. Thus, we examined whether Chk1i can potentiate the potency of PLX4032 in melanoma. We treated the melanoma cells with Chk1i (PF477736, 0.15 μM) in combination with increasing concentrations of BRAFi (PLX4032, 0–20 μM). At this low concentration of PF477736, the growth of both A375 and A375-PLX-R cells is only slightly inhibited (Figure 3). Our results indicate that the cell viability of A375 cells is only synergistically inhibited by 0.15 μM PF477736 and high concentrations (12.5–20 μM) of PLX4032 (Figure 4A). However, both inhibitors have synergy in inhibiting the cell viability of A375-PLX-R cell at all tested PLX4032 concentrations (Figure 4B). In colony formation assay, small synergy of PF477736 and PLX4032 was observed with A375 cell (Figure 4C). In contrast to cell viability, A375-PLX-R cells form more colonies in the presence of 2.5–15 μM PLX4032 than in its absence (compare Figure 4B and 4D, green curves), indicating A375-PLX-R cells are addicted to PLX4032 during 10-day colony formation culture. Strikingly, there are fewer colonies in PF477736+PLX4032 than in PF477736 treatment alone at all tested PLX4032 concentrations (Figure 4D, purple curve). Representatives of colony formation of Figure 4C and 4D are shown in Figure 5A and 5B. Data from more than three experiments with 10 μM of PLX4032 and 0.15 μM of PF477736 are shown in Figure 5C. This result indicates that the PLX4032-resistant A375-PLX-R cell are sensitized to PLX4032 in the presence of PF477736 or that A375-PLX-R cell are more sensitive to PF477736 in the presence of PLX4032.

**Figure 6:**
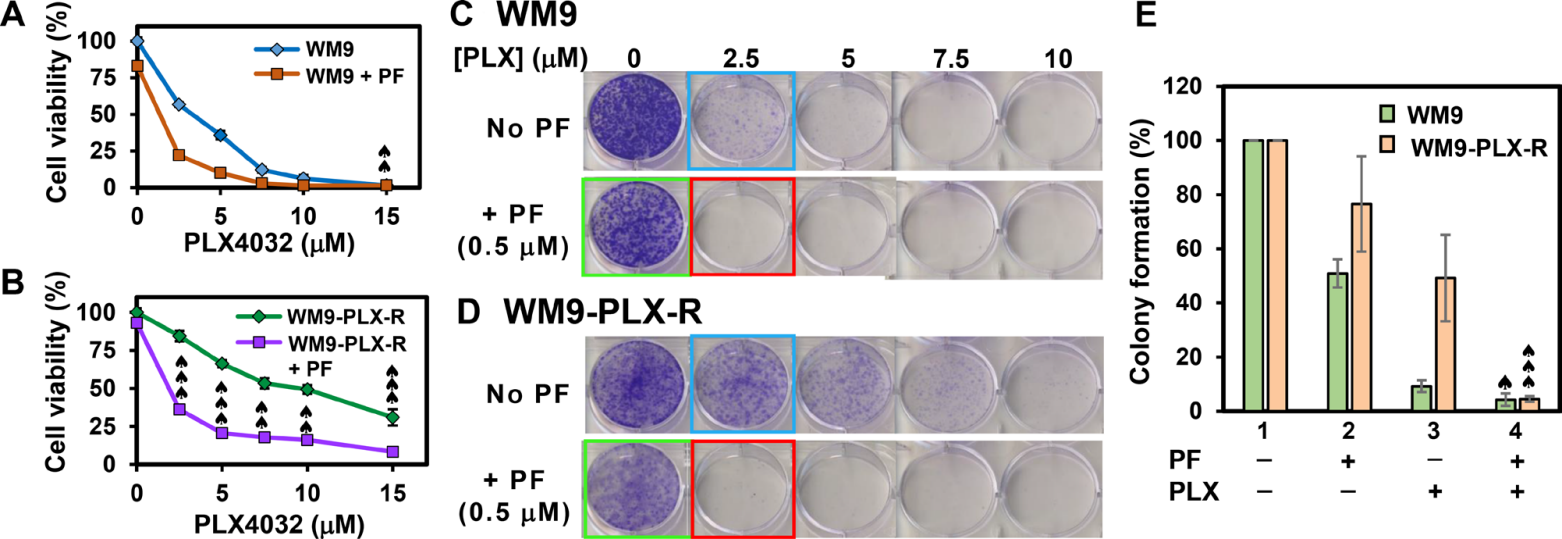
Cell viability and colony formation of WM9 and WM9-PLX-R cells in the presence of BRAFi PLX4032 and/or Chk1i PF477736 The melanoma cells were treated with Chk1i (PF477736, 0.5 μM) in combination with increasing concentrations of BRAFi (PLX4032, 0–15 μM) as indicated on the X-axis. Cell viability of WM9 (**A**) and WM9-PLX-R (**B**) cells as well as colony formation of WM9 (**C**) and WM9-PLX-R (**D**) cells are similar to those in Figure 4. Top panels in both (C) and (D) are cells treated with different concentrations of PLX4032 as indicated and bottom panels are cells treated with respective PLX4032 concentrations plus 0.5 μM PF477736. Data from more than three experiments with 2.5 μM of PLX4032 (blue boxed), 0.5 μM of PF477736 (green boxed), and PLX4032 + PF477736 (red boxed) are shown in (**E**). The error bars, CI calculation, and ♠ symbol were similar to those as described in Figure 4.

Combinatorial treatments with PF477736 (0.5 μM) and PLX4032 (2.5–10 μM) also synergistically inhibit the cell viability of WM9-PLX-R cells (Figure 6B), but not of WM9 cells (Figure 6A). In the colony formation assay, small synergy of PF477736 and PLX4032 was observed with WM9 cell (Figure 6C and Figure 6E). In contrast to A375-PLX-R cells, WM9-PLX-R cells show no addiction to PLX4032 during 10-day colony formation culture (compare Figure 5B and Figure 6D). However, similar to A375-PLX-R cells, WM9-PLX-R cells regain the sensitivity to PLX4032 in the presence of PF477736 for all tested PLX4032 concentrations (Figure 6D and Figure 6E). Taken together, results from our studies indicate that combinatorial treatments with BRAFi and Chk1i synergistically reduce cell viability and colony formation of PLX4032-resistant melanoma cells. This indicates that Chk1 inhibitors can re-sensitize BRAFi-resistant melanoma cells to BRAFi.

### Co-treatment with PLX4032 and PF477736 suppresses tumor formation

**Figure 7:**
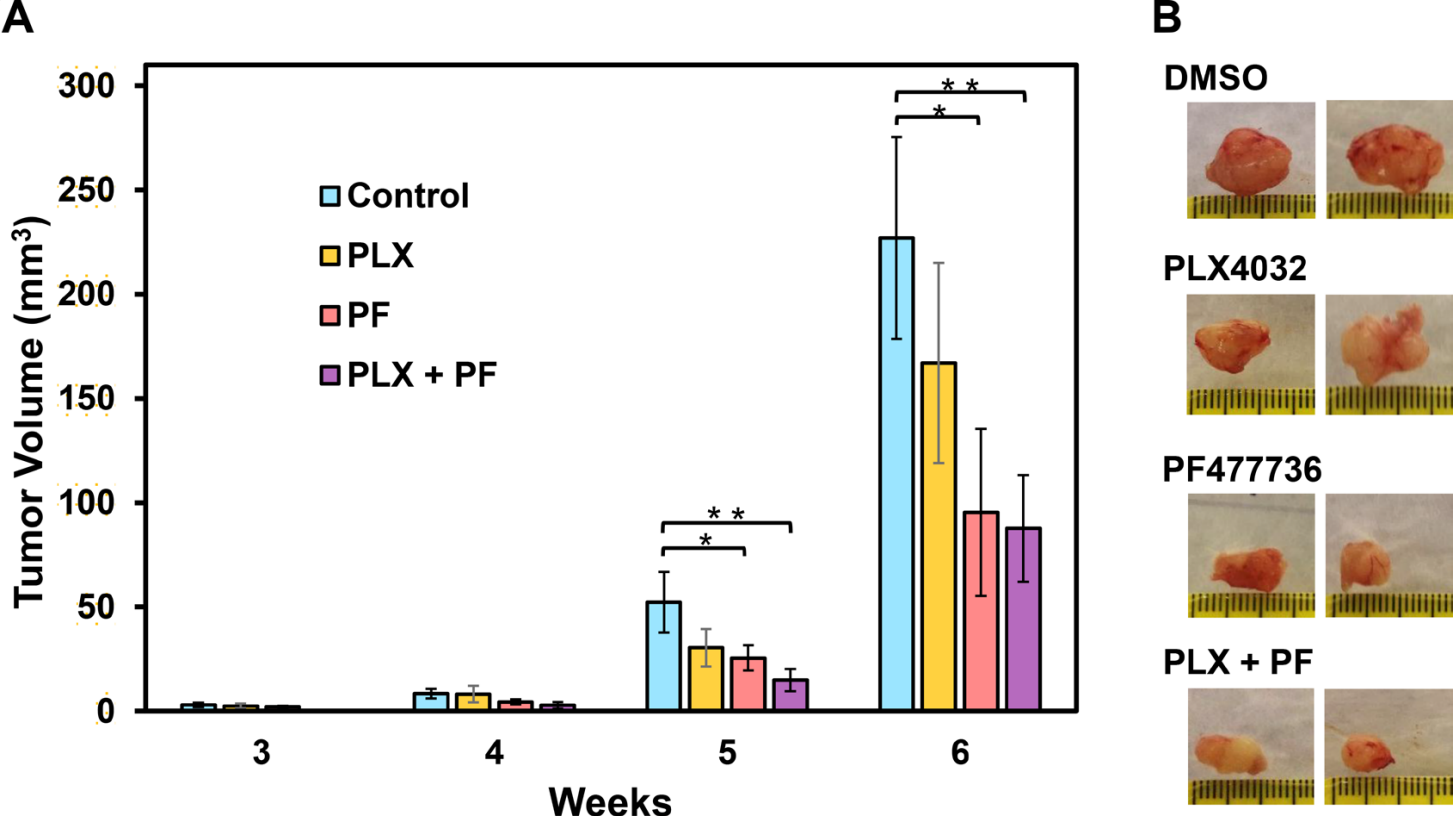
Chk1 inhibitor PF477736 reduces melanoma tumor growth Tumor xenografts of A375-PLX-R melanoma spheroid cells were injected in each front flank of female nude mice. Drug treatments were initiated at the time of tumor cell injection. The mice were injected with 15 mg/Kg body weight of Chk1i (PF477736) or DMSO for three days per week and/or 20 mg/Kg body weight of PLX4032 by oral gavage. Tumor volume were measured every week. (**A**) The mice receiving PF477736 alone and PF477736+PLX4032, but not PLX4032 alone, have substantially smaller tumor than the controls after week 5 and week 6 with co-treatment showing more significance. The error bars reported are the standard deviations of the averages from six tumors and the *P*-value was calculated by Student's *t*-test. One star indicates *P* < 0.1 and two stars indicate *P* < 0.05. (**B**) Two representative tumors from each treatment group are shown.

We next examined the influence of Chk1i on the ability of melanoma cancer stem (MCS) cells (selected as spheroids on ultra-low attachment dishes) to form tumors on female nude mice. We select A375-PLX-R MCS for tumor studies because they are the most aggressive cells [40]. We injected 100,000 A375-PLX-R MCS cells into each front flank of nude mice and monitored tumor formation with drug treatments initiated at the time of tumor cell injection. Figure 7 shows that tumors appear at 3-week post-injection. The mice receiving PF477736 alone and PF477736+PLX4032, but not PLX4032 alone, have substantially smaller tumors than the controls after week 5 and week 6 that is more significant in co-treated tumors. This finding suggests that Chk1 inhibition may be an effective anticancer strategy in PLX4032-resistant melanoma.

### Chk1 inhibition regulates Chk1 phosphorylation

In DNA damage response, Chk1 arrests cell cycle progression following genotoxic stress and stalled replication to prevent the entry of cells with damaged DNA into mitosis [11]. In addition to its role in the DNA damage response, Chk1 also controls the initiation of DNA replication, stabilization of replication forks, and coordination of mitosis and DNA repair [24]. It is unclear whether Chk1i disrupts one or all of these pathways to inhibit melanoma growth. It is also not clear why PLX4032-resistant melanoma cells regain the sensitivity to PLX4032 in the presence of Chk1i (Figures 4–7). Thus, we attempted to reveal the underlying mechanisms.

**Figure 8:**
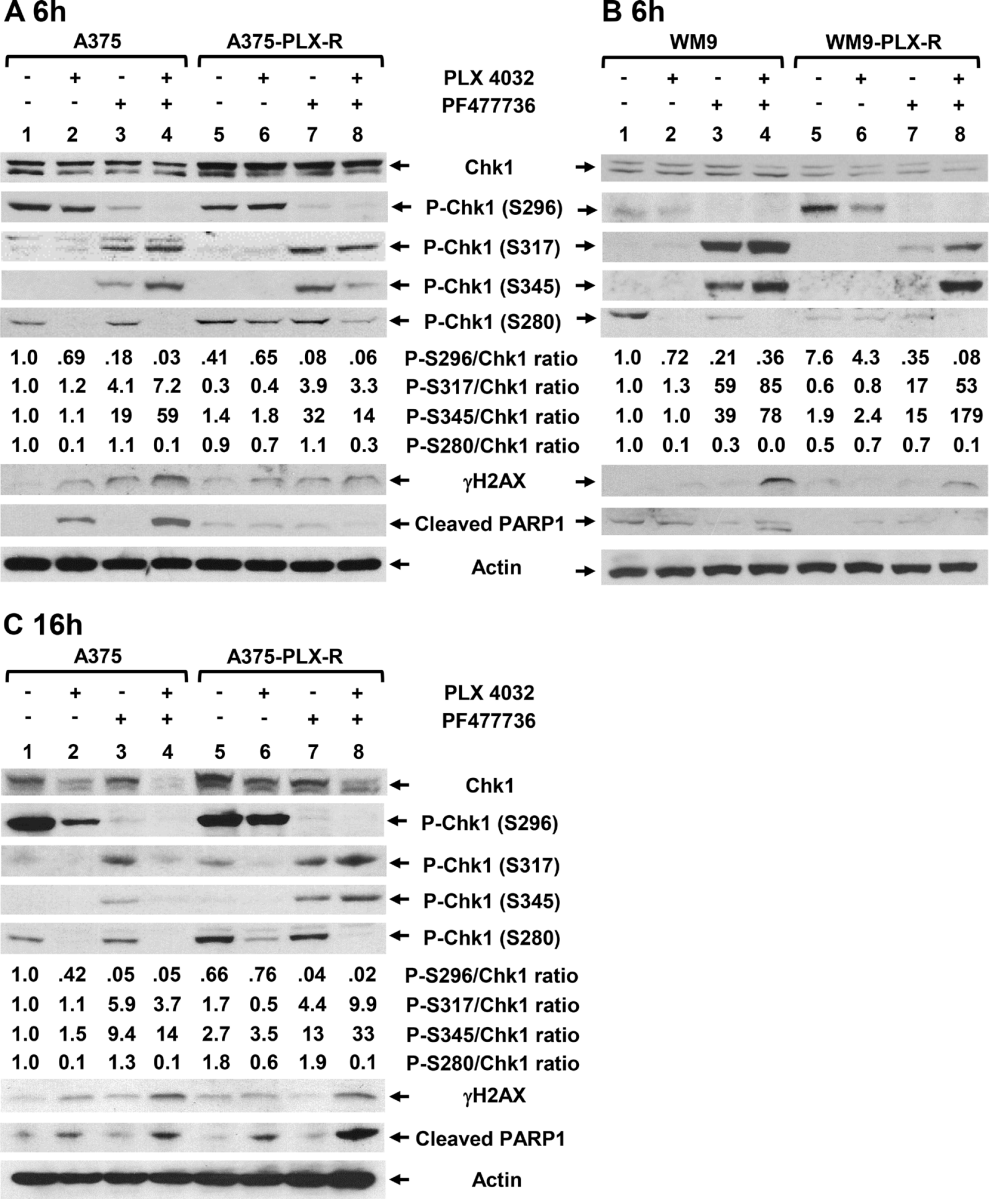
Expression of DNA damage response proteins in melanoma cells following treatments with BRAFi and/or Chk1i Cells were treated with PLX4032 (7.5 μM) and/or PF477736 (0.15 μM) for 6 h or 16 h. (**A**) A375 and A375-PLX-R cells treated with drugs for 6 h; (**B**) WM9 and WM9-PLX-R cells treated with drugs for 6 h; and (**C**) A375 and A375-PLX-R cells treated with drugs for 16 h. Western blotting analyses with respective antibodies were performed. Ratios of phosphorylated Chk1 proteins at S296, S317, S345, or S280 over total Chk1 protein were calculated and then adjusted relative to the value of lane 1 (untreated A375 or WM9 cells) as described in Figure 2. Relative ratios are the averages of two sets of images from two independent experiments.

Chk1 is phosphorylated at S317 and S345 by ATR protein kinases in response to DNA damage to regulate cell cycle progression [41, 42]. After phosphorylation by ATR at S317 and S345, Chk1 can autophosphorylate itself at S296 [15, 16]. Surprisingly, it has been reported that inhibition of Chk1 kinase activity leads to hyper-phosphorylation of Chk1 at S317 and S345 in cancer cells [31, 43]. It has been shown that ATR-mediated Chk1 phosphorylation is antagonized by a Chk1-regulated protein phosphatase 2A circuit [43]. Hyper-phosphorylation of Chk1 at S317 and S345 has been used as an indicator of Chk1 inhibition [31]. Thus, we determined the levels of Chk1 phosphorylation in the four melanoma cells following transient treatments with PLX4032 (7.5 μM) and/or PF477736 (0.15 μM) for 6 h. Western blotting analyses indicate that the total Chk1 protein level is increased in untreated A375-PLX-R cells (Figure 8A) but decreased in untreated WM9-PLX-R cells (Figure 8B) as compared to their parental cell lines. Drug treatments only slightly alter the total Chk1 protein levels in all four cell lines. The level of Chk1 phosphorylation at S296 is greatly reduced when all four cell lines are treated with PF477736 or PLX4032+PF477736, indicating that PF477736 blocks Chk1 activity *in vivo* (Figure 8A, see ratios of P-S296/Chk1). When A375, WM9, and WM9-PLX-R cell lines are treated with PLX4032 alone, the level of Chk1 phosphorylation at S296 is slightly reduced. Our data indicate that the levels of Chk1 phosphorylation at S296 is reversely proportional to the levels of Chk1 phosphorylation at S317 and S345 (Figure 8A and 8B). The levels of Chk1 Ser317 and S345 phosphorylation are elevated when all four cell lines are treated with PF477736 or PLX4032+PF477736, but not with PLX4032 single treatment (Figure 8A and 8B, see ratios of P-S317/Chk1 and P-S345/Chk1). Combinatorial treatments with PLX4032 and PF477736 for 6 h induced the highest extents of Chk1 S317 and S345 phosphorylation in A375, WM9, and WM9-PLX-R cells, but not in A375-PLX-R cells (Figure 8A and 8B, see ratios of P-S317/Chk1 and P-S345/Chk1).

To study the duration of drug effects, we also determined Chk1 alterations in A375 and A375-PLX-R cells following treatments with PLX4032 (7.5 μM) and/or PF477736 (0.15 μM) for 16 h. Compared to 6 h treatments, the total Chk1 protein levels are substantially reduced in both cell lines following treatments with PLX4032 and PLX4032+PF477736 for 16 h (Figure 8C, Chk1 lanes 1–8). Thus, PLX4032 treatment can reduce Chk1 protein expression and its effect is enhanced by the presence of PF477736. Similar to 6 h treatment, the level of Chk1 phosphorylation at S296 is greatly reduced when A375 and A375-PLX-R cells are treated with PF477736 or PLX4032+PF477736 (Figure 8C, see ratios of P-S296/Chk1). When A375 cell lines are treated with PLX4032 alone, the level of Chk1 phosphorylation at S296 is also reduced. The levels of Chk1 S317 and S345 phosphorylation are induced most in PLX4032+PF477736-treated A375-PLX-R cells (Figure 8C, see ratios of P-S317/Chk1 and P-S345/Chk1). Thus, the phosphorylation levels of Chk1 at S317 and S345 in A375-PLX-R cells are higher at 16 h than 6 h following combinatorial treatment with PLX4032 and PF477736 (compare Figure 8A and 8C).

Next, we examined the drug effects on Chk1 phosphorylation at S280 which is carried out by p90 RSK at G0/G1 transition [19] or by AKT1 in response to DNA damage during the G2 phase [20–22]. Chk1 phosphorylation at S280 by these Ras downstream kinases alters Chk1 localization between the cytoplasm and the nucleus and affects checkpoint activation. The level of Chk1 S280 phosphorylation is unchanged in untreated A375-PLX-R cells (Figure 8A) but decreased in untreated WM9-PLX-R cells as compared to their parental cell lines (Figure 8B). The levels of Chk1 S280 phosphorylation are diminished when A375 and WM9 cell lines are treated with PLX4032 or PLX4032+PF477736 for 6 h (Figure 8A and 8B). However, the levels of Chk1 S280 phosphorylation in A375-PLX-R and WM9-PLX-R cell lines are substantially reduced only following PLX4032+PF477736 treatment for 6 h (Figure 8A and 8B, see ratio of P-S280/Chk1). The reduction of Chk1 S280 phosphorylation is more pronounced when A375-PLX-R cells are treated with PLX4032 or PLX4032+PF477736 for 16 h (Figure 8C, see ratio of P-S280/Chk1). Combinatorial treatments to PLX4032-resistant cells synergistically reduce Chk1 S280 phosphorylation. For example, the ratios of P-S280/Chk1 are 1.8, 0.6, 1.9, and 0.1 in untreated, PLX4032-treated, PF477736-treated, and double-treated A375-PLX-R cells. Taken together, combinatorial treatments with PLX4032 and PF477736 reduce the levels of total Chk1 protein and Chk1 phosphorylation at S280 and S296, but increases the levels of Chk1 phosphorylation at S317 and S345 in both PLX4032 sensitive and resistant melanoma cells.

### Chk1 inhibition induces DNA damage and apoptosis

To further investigate the mechanisms by which Chk1i boosts BRAFi sensitivity in BRAFi-resistant melanoma, we analyzed parameters of DNA damage and apoptosis. To assess the effect of Chk1i on DNA damage, we measured the levels of phosphorylated H2AX (γH2AX) which is an indication of stalled replication forks and double-strand breaks [44, 45]. The levels of γH2AX are elevated when A375 and WM9 cell lines are treated with PLX4032 or PF477736 individually and are further enhanced following PLX4032+PF477736 treatment for 6 h (Figure 8A and 8B). Following treatments with PLX4032+PF477736 for 6 h, A375-PLX-R and WM9-PLX-R cell lines also induce γH2AX, but at a lower extent as compared with their parental cell lines. The patterns of γH2AX in A375 and A375-PLX-R cells following 16 h drug treatments are similar to those following 6 h drug treatments (compare Figure 8A and 8C) except the γH2AX level is slightly lower in PF477736-treated than untreated A375-PLX-R cells. Nevertheless, the level of γH2AX is synergistically elevated when all melanoma cell lines are treated by PLX4032+PF477736.

To monitor the effect of Chk1i on apoptosis, we measured the levels of cleaved PARP1, a hallmark of caspase-dependent apoptosis [46]. The level of cleaved PARP1 of A375 cells is elevated following PLX4032 treatment for 6 h and is further enhanced following PLX4032+PF477736 treatment, but is not increased by PF477736 single treatment (Figure 8A). There are little changes in the levels of cleaved PARP1 when A375-PLX-R, WM9, and WM9-PLX-R cells are treated with PLX4032 and/or PF477736 for 6 h. However, the levels of cleaved PARP1 of A375-PLX-R cells are elevated following PLX4032 treatment and is further enhanced following PLX4032+PF477736 treatment for 16 h (Figure 8C). This suggests that induction of caspase-dependent apoptosis is delayed in PLX4032- and PLX4032+PF477736-treated A375-PLX-R cells. PF477736 single treatments for either 6 h or 16 h to all four cell lines do not induce PARP1 cleavage (Figure 8). Taken together, these results indicate that combinatorial treatment with PLX4032 and PF477736 induces DNA damage and cell death pathways in melanoma cells.

## DISCUSSION

BRAF inhibitor PLX4032 (vemurafenib) is very effective in treating malignant melanoma patients with BRAF^V600E^ mutation (reviewed in [7]). However, response to PLX4032 is not durable because many patients acquire drug resistance [9, 10]. In this report, we have developed a novel strategy to overcome such resistance. Cell cycle checkpoints are favorable targets for anticancer therapies because they control cancer cell responses to chemotherapy and radiation [23, 24]. Although Chk1 inhibitors have been demonstrated to reduce the cell viability of melanoma cells [32–34], the efficacy of Chk1 inhibitors in melanoma therapy required further studies. Here, we show that a specific Chk1i PF477736 can overcome the resistance to PLX4032 of PLX4032-resistant melanoma cells. Although PF477736 has 100-fold specificity to Chk1 over Chk2 [28, 30], we cannot rule out it may also inhibit Chk2 in our assays. Our results demonstrate that PF477736 and PLX4032 can synergistically inhibit the cell viability and colony formation of PLX4032-resistant melanoma cells. Moreover, Chk1i and combined Chk1i+BRAFi can reduce the growth of BRAFi-resistant melanoma tumor in mouse xenografts. Currently, the agents used in combination with Chk1i to treat cancers are all antimetabolites (such as cisplatin and gemcitabine) that damage DNA or interfere with DNA synthesis. Our results represent the first successful application using Chk1i and a non-antimetabolite BRAFi. The significance of our studies is justified by our Kaplan-Meier analyses (Figure 1) of more than 40 melanoma patients, indicating that low mRNA expression of Chk1 is significantly associated with good overall survival of melanoma patients. Thus, Chk1 inhibitors combined with BRAFi may provide anticancer therapeutics to current treatment regimens for BRAFi-resistance melanoma patients.

We have tested the effect of Chk1i on two melanoma cell lines (A375 and WM9) and their derived PLX4032-resistant cell lines. The two PLX4032-resistant cell lines, which are p53 positive but have different PTEN status, develop resistance to PLX4032 through different mechanisms. AKT is constitutively activated in A375-PLX-R but not in WM9-PLX-R cells. After transient treatment with 7.5 μM PLX4032 for 6 h, ERK pathway is completely inhibited in A375-PLX-R cells but is partially active in WM9-PLX-R cells. We notice that our data is different from the previous report [40] showing that level of phosphorylated ERK remains high when the same A375-PLX-R cell line is treated with 1 μM PLX4032 for 24 h. The discrepancy may be caused by different PLX4032 concentrations and duration of treatments. Because A375-PLX-R cells were developed by gradual increase of PLX4032 concentrations up to 4 μM and were maintained with medium containing 1 μM PLX4032, PLX4032 at 1 μM may be not sufficient to inhibit ERK phosphorylation. We also show that the dependence of Chk1 function for survival is different between PLX4032-resistant lines and their parental A375 and WM9 cell lines. PF477736 is more effective in inhibiting cell viability and colony formation of A375-PLX-R than A375 cells. However, PF477736 is more effective in inhibiting cell viability and colony formation of WM9 than WM9-PLX-R cells. Although the mechanism behind this difference requires further investigation, the different PTEN status and activation of alterative survival pathways may contribute to their differential response to Chk1i treatment. It has been shown that PTEN plays a direct role in DNA damage response through interaction with Chk1 and the tumor suppressor p53 [39]. Because all four cell lines are p53 positive, the differential response to Chk1i in A375-PLX-R and WM9-PLX-R is likely independent of p53, even though several studies have shown that the response to Chk1i is dependent on p53 [24, 33, 34]. Strikingly, although A375-PLX-R and WM9-PLX-R cells develop resistance to PLX4032 through different mechanisms, they re-sensitize to PLX4032 in the presence of PF477736.

To reveal why Chk1i can trigger BRAFi resistant cells regaining BRAFi sensitivity, we detect that Chk1i treatments inhibit auto-phosphorylation of Chk1 at S296 but induce hyper-phosphorylation of Chk1 at S317 and S345 which is mediated by ATR in response to DNA damage [41, 42]. The reduction of Chk1 phosphorylation at S296 in cells treated with PF477736 indicates that the inhibitor blocks Chk1 activity *in vivo*. Hyper-phosphorylation of Chk1 at S317 and S345 has been used as an indicator of Chk1 inhibition [31, 43]. Indeed, our data indicate that the levels of Chk1 phosphorylation at S317 and S345 are reversely proportional to the level of Chk1 phosphorylation at S296 (Figure 8). It has been demonstrated that ATR-mediated Chk1 phosphorylation is antagonized by a Chk1-regulated protein phosphatase 2A circuit [43]. Thus, when Chk1 activity is inhibited by PF477736, PP2A is inactive to dephosphorylate Chk1, leading to hyper-phosphorylation of Chk1. Combinatorial treatments with PLX4032 and PF477736 to A375, WM9, WM9-PLX-R cells for 6 h induce the highest levels of Chk1 S317 and S345 phosphorylation (Figure 8A and 8B). However, A375-PLX-R cells contain the highest levels of Chk1 S317 and S345 phosphorylation after combinatorial treatment with PLX4032 and PF477736 for 16 h but not for 6 h (compare Figure 8A and 8C). This suggests that A375-PLX-R cells require extended Chk1i+BRAFi treatment to induce Chk1 hyper-phosphorylation and associated cell death as indicated with high level of cleaved PARP1 in Figure 8C. Because hypo-phosphorylation at S296 and hyper-phosphorylation at S317 and S345 of Chk1 can serve as indicators of Chk1 inhibition [31], combined treatment with PLX4032 and PF477736 is more effective than PF477736 treatment alone to inhibit Chk1 activity.

We also show that PLX4032 treatments to BRAFi-sensitive cells severely inhibit Chk1 S280 phosphorylation which can be mediated by two Ras downstream kinases p90 RSK [19] or AKT1 [21]. PF477736 enhances PLX4032 to inhibit Chk1 S280 phosphorylation in BRAFi-resistant cells (Figure 8). Because the levels of phosphorylated AKT are increased (Figure 2) but the levels of Chk1 S280 phosphorylation are substantially reduced (Figure 8) in PLX4032-treated A375, A375-PLX-R, and WM9 cells, we suggested AKT plays limited role in Chk1 S280 phosphorylation in these melanoma cells. Synergistic effects exist with PLX4032 and PF477736 treatments in reducing the levels of total Chk1 protein and Chk1 S280 phosphorylation and increasing the levels of Chk1 S317 and S345 phosphorylation. Lee *et al.* [31] have found that Ras signaling engages Chk1 in a subset of osteosarcoma, ovarian, and breast cancer cells to enable their survival upon DNA damage, irrespective of p53 mutation status. The authors demonstrated a feedback mechanism between Ras signaling and Chk1 function: Ras drives Chk1 expression and Chk1 prevents hyper-activation of Ras signaling to sustain cancer cell proliferation. Thus, we suggest that the synergistic effects of combinatorial PLX4032 and PF477736 treatments on Chk1 expression and phosphorylation may perturb cell cycle progression, induce DNA damage, and lead to cell death.

The above scenario is supported by the elevated levels of γH2AX and cleaved PARP1 in A375 cells treated with PLX4032+PF477736. Although the level of γH2AX is high but the level of cleaved PARP1 does not increased when A375-PLX-R, WM9, and WM9-PLX-R cells are treated with PLX4032+PF477736 for 6 h. PARP1 cleavage is induced in A375-PLX-R cells only after 16 h treatments with PLX4032 and PLX4032+PF477736. This implies that A375-PLX-R cells require extended treatments with PLX4032 and PLX4032+PF477736 to induce caspase-dependent apoptosis. PF477736 treatments to A375 for 6 h and A375-PLX-R cells for 16 h does not induce PARP cleavage, but can potentiate the stimulation action of PLX4032 on PARP1 cleavage (Figure 8A and 8C). Because PF477736 treatment alone reduces cell viability and colony formation of all four cell lines (Figure 3), we suggest that PF477736 may induce caspase-independent cell death pathways [47]. Our result is similar to the findings that Chk1i do not increase the levels of γH2AX and activated caspase 3 in K-Ras-induced pancreatic adenocarcinomas [48]. However, Chk1 has been shown to suppress caspase-dependent apoptotic pathways [49, 50] and PARP cleavage is detected in melanoma cells treated with Chk1 inhibitor AR323 for longer than 24 h [32]. In conclusion, our results indicate that combinatorial treatment with PLX4032 and PF477736 promotes DNA damage and may induce different pathways of cell death in melanoma cells.

**Figure 9:**
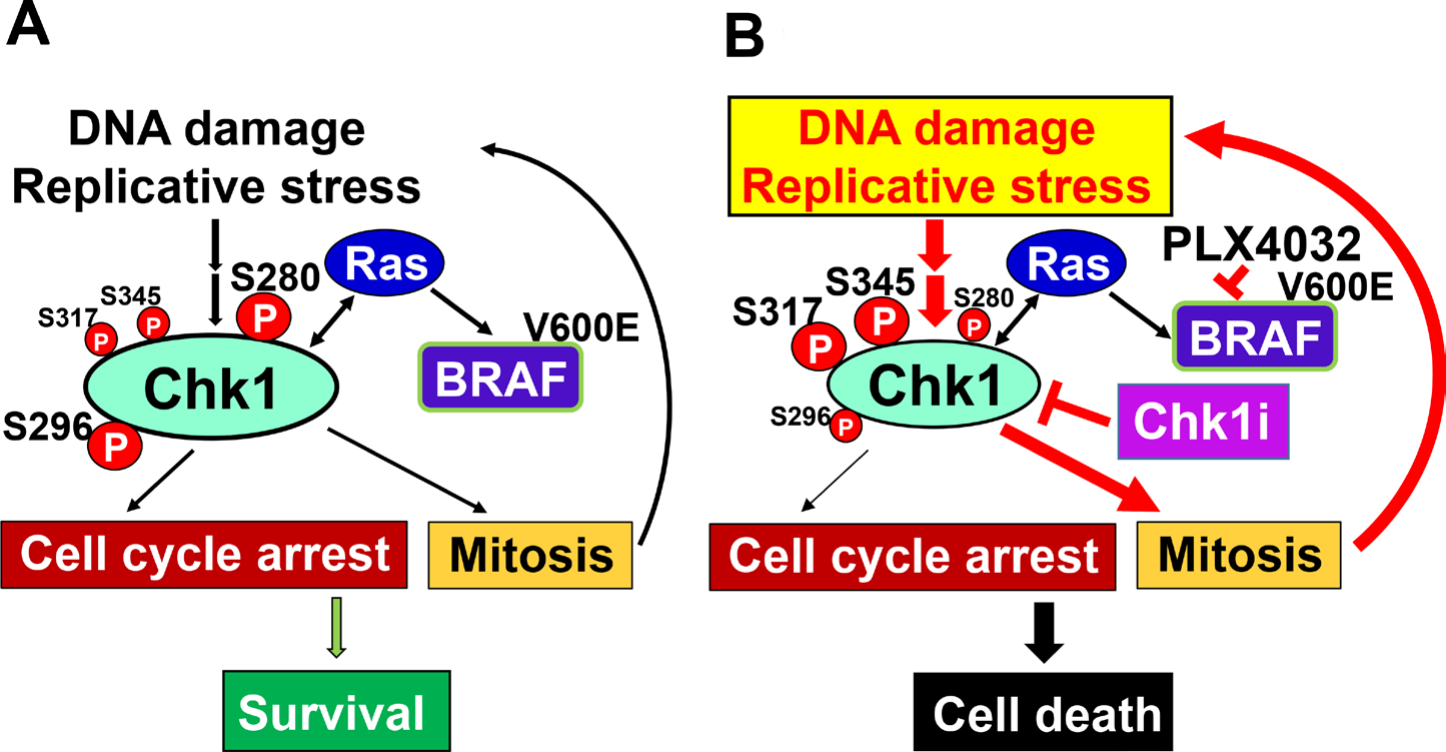
Model for Chk1 regulation of cancer cell survival (**A**) Cancer cells use DNA damage response to manage Ras-induced DNA damage and replication stress. Ras and Chk1 use feedback pathways to minimalize DNA damage, maintain moderate cell cycle arrest, and sustain cell proliferation. The levels of total Chk1 and Chk1 phosphorylation at S280 and S296 are high, while the levels of Chk1 phosphorylation at S317 and S345 are low. (**B**) Chk1i in combination with BRAFi could lead cancer cells to arrest improperly, enter into mitosis with highly damaged DNA, and undergo apoptosis. Inhibition of Chk1 can decrease the levels of total Chk1 and Chk1 phosphorylation at S280 and S296, but increase Chk1 phosphorylation at S317 and S345.

Although the mechanisms by which Chk1 inhibitors boost BRAFi sensitivity in BRAFi-resistant melanoma still need further clarification, our results support a model that Chk1 inhibition in combination with BRAFi can lead melanoma cells to arrest improperly, enter into mitosis with highly damaged DNA, and undergo mitotic catastrophe/cell death (Figure 9). This model has been suggested in combinatorial treatments of Chk1i with DNA damaging agents to cancer cells [32, 51]. Cancer cells induce moderate DNA damage response to manage Ras-induced DNA damage and replication stress. The levels of total Chk1 and Chk1 phosphorylation at S280 and S296 are high, while the levels of Chk1 phosphorylation at S317 and S345 are low. In the presence of Chk1i and BRAFi, cancer cells contain high levels of damaged DNA and replicative stress but lose cell cycle checkpoints. The levels of total Chk1 and Chk1 phosphorylation at S280 and S296 are decreased, but the levels of Chk1 phosphorylation at S317 and S345 are increased. Our work suggests that combinatorial therapies with Chk1i and BRAFi will improve the outcomes and curtail the resistance to BRAFi in melanoma.

## MATERIALS AND METHODS

### Melanoma cell lines and culture

A375 and its PLX4032-resistant cell lines were maintained under attached conditions in growth media containing DMEM (Invitrogen, Frederick, MD, USA) supplemented with 4.5 mg/ml D-glucose, 2 mM L-glutamine, 1 mM sodium pyruvate, and 5% fetal calf serum. WM9 and its PLX4032-resistant cell lines were grown in 2% Tumor Medium (Tu2%) containing a 4:1 mixture of MCDB 153 medium and Leibovitz's L15 medium supplemented with 0.005 mg/ml bovine insulin, 1.68 mM CaCl_2_, and 2% fetal bovine serum. PLX4032-resistant A375-PLX-R cell line has been created by gradual increase of PLX4032 up to 4 μM as reported [40]. WM9-PLX-R cell line was derived from WM9 cell line similarly according to the procedure described [40]. Briefly, cells were treated with 1 μM PLX4032 in monolayer culture in growth medium for several weeks. The surviving cells were then expanded and cultured in increasing doses of PLX4032 up to 4 μM. The resulting PLX4032-resistant cells were routinely maintained with media containing 1 μM PLX4032. Spheroid melanoma cells were derived from monolayer cultures as described previously [40].

### Western blotting

These antibodies used for Western blotting were purchased from Cell Signaling Technology: ERK1/2, T202Y204 phosphorylated ERK1/2, AKT, S473 phosphorylated AKT, S296 phosphorylated Chk1, S317 phosphorylated Chk1, S345 phosphorylated Chk1, γH2AX, and cleaved PARP. Antibody of Chk1 is from Bethyl Laboratories Inc. Antibodies of β-actin and horseradish peroxidase-conjugated anti-mouse/anti-rabbit antibodies are from Sigma-Aldrich and Bio-Rad, respectively. Cell extracts were prepared as described [52] and about 25 μg of total protein were separated on 10–16% SDS-polyacrylamide gels and transferred to nitrocellulose membranes for Western blotting [52]. To calculate the ratios of phosphorylated proteins over total proteins, the images from the X-ray films were scanned and quantitated by ImageQaunt software (GE Healthcare Life Sciences). After subtracting the background, the calculated ratios were then adjusted relative to the ratio value of untreated A375 cells.

### Cell viability and colony formation assays

Cell viability was measured using the neutral red uptake assay [53]. Melanoma cells were seeded in 12-well flat bottom tissue culture plates. One-day post-seeding, the cells were treated with PLX4032 (APEXBIO), PF477736 (Sigma-Aldrich), or DMSO for 48 h. The cells were then recovered in regular media for 2–3 days. The plates were incubated for 2 h in regular medium containing 40 μg/ml of neutral red (3-amino-7-dimethylamino-2-methyl-phenazine hydrochloride, Sigma). After the cells being washed with PBS, the dye was extracted from each well with acidified ethanol solution and the absorbance at 540 nm was read by a Multiskan Spectrum microplate spectrometer (Thermo Lab systems).

For clonogenic survival assays, cells were seeded at 5000 cells per well in 6-well plates and treated with drugs as described above. Regular media was replaced after treatment. After 10 days, cells were stained with 0.5% crystal violet in 20% methanol and counted.

Combination index (CI) for cells treated with two drugs was determined by CompuSyn software for the Chou and Talalay analysis as described [54]. A CI value less than 1 indicates synergic effect.

### Tumor xenograft studies

Cells were grown for ten days as spheroids and a single cell suspension, prepared by trypsin digestion, was resuspended in phosphate buffered saline containing 30% Matrigel and 100 μl containing 0.1 million cells were injected subcutaneously at the two front flanks of NOD/scid/IL2 receptor gamma-knockout mice (NSG mice) using a 26.5-gauge needle. Three mice were used per data point with two tumors per mouse. PLX4032 was dissolved in PBS containing 10% DMSO and 100 μl was delivered by oral gavage (20 mg/Kg body weight) three times per week (M/W/F) beginning at the time of tumor cell injection. PF477736 dissolved in DMSO was mixed with 40% PEG 400 in PBS and Tween 80 at 2:97:1 ratio and 15 mg/Kg was delivered by intraperitoneal injection three times per week (M/W/F). Tumor growth was monitored by measuring tumor diameter and calculating tumor volume using the formula, volume = 4/3π × (radius)^3^. Mice were euthanized by injecting 250 μl of a 2.5% stock of avertin per mouse followed by cervical dislocation, and tumor samples were harvested for morphological assessment. These experiments were reviewed and approved by the University of Maryland–Baltimore Institutional Animal Care and Use Committee.

## Abbreviations

A375-PLX-R cell line: a PLX4032 resistant cell line from A375
AKT/PKB: protein kinase B
ATR: ataxia telangiectasia and Rad3-related protein
BRAF: v-Raf murine sarcoma viral oncogene homolog B1
BRAFi: BRAF inhibitors
CI: combination index
Chki: Chk1 inhibitors
ERK: extracellular signal-regulated kinase
HR: hazard ratio
IC_50_: the concentration of drug to inhibit 50% of cell growth
MEK: mitogen-activated protein kinase
MCS: melanoma cancer stem cell
p90 RSK: Ras/mitogen-activated 90-kDa ribosomal S6 kinase
PI3K: phosphatidylinositol 3-kinase
PDK1: 3–phosphoinositide-dependent protein kinase-1
PF477736: a specific inhibitor of Chk1
PLX4032 (Vemurafenib): a specific BRAF inhibitor
PTEN: phosphatase and tensin homology
T_1/2_: the time required for 50%of the patients remain alive
ΔT_1/2_: the difference between two T_1/2(s)_ for each group of patients
V600E mutation: residue 600 Valine to Asparagine mutation
WM9-PLX-R cell line: a PLX4032 resistant cell line from WM9
γH2AX: phosphorylated histone H2AX.
